# From Wolves to Dogs, and Back: Genetic Composition of the Czechoslovakian Wolfdog

**DOI:** 10.1371/journal.pone.0143807

**Published:** 2015-12-04

**Authors:** Milena Smetanová, Barbora Černá Bolfíková, Ettore Randi, Romolo Caniglia, Elena Fabbri, Marco Galaverni, Miroslav Kutal, Pavel Hulva

**Affiliations:** 1 Faculty of Tropical AgriSciences, Czech University of Life Sciences Prague, Prague, Czech Republic; 2 Laboratorio di Genetica, Istituto Superiore per la Protezione e Ricerca Ambientale (ISPRA), Ozzano Emilia (BO), Italy; 3 Department 18/Section of Environmental Engineering, Aalborg University, Aalborg, Denmark; 4 Institute of Forest Ecology, Faculty of Forestry and Wood Technology, Mendel University in Brno, Brno, Czech Republic; 5 Friends of the Earth Czech Republic, Olomouc branch, Olomouc, Czech Republic; 6 Department of Zoology, Charles University in Prague, Prague, Czech Republic; 7 Department of Biology and Ecology, Ostrava University, Ostrava, Czech Republic; Ben-Gurion University of the Negev, ISRAEL

## Abstract

The Czechoslovakian Wolfdog is a unique dog breed that originated from hybridization between German Shepherds and wild Carpathian wolves in the 1950s as a military experiment. This breed was used for guarding the Czechoslovakian borders during the cold war and is currently kept by civilian breeders all round the world. The aim of our study was to characterize, for the first time, the genetic composition of this breed in relation to its known source populations. We sequenced the hypervariable part of the mtDNA control region and genotyped the Amelogenin gene, four sex-linked microsatellites and 39 autosomal microsatellites in 79 Czechoslovakian Wolfdogs, 20 German Shepherds and 28 Carpathian wolves. We performed a range of population genetic analyses based on both empirical and simulated data. Only two mtDNA and two Y-linked haplotypes were found in Czechoslovakian Wolfdogs. Both mtDNA haplotypes were of domestic origin, while only one of the Y-haplotypes was shared with German Shepherds and the other was unique to Czechoslovakian Wolfdogs. The observed inbreeding coefficient was low despite the small effective population size of the breed, possibly due to heterozygote advantages determined by introgression of wolf alleles. Moreover, Czechoslovakian Wolfdog genotypes were distinct from both parental populations, indicating the role of founder effect, drift and/or genetic hitchhiking. The results revealed the peculiar genetic composition of the Czechoslovakian Wolfdog, showing a limited introgression of wolf alleles within a higher proportion of the dog genome, consistent with the reiterated backcrossing used in the pedigree. Artificial selection aiming to keep wolf-like phenotypes but dog-like behavior resulted in a distinctive genetic composition of Czechoslovakian Wolfdogs, which provides a unique example to study the interactions between dog and wolf genomes.

## Introduction

Recent genomic studies indicate that dogs were domesticated via a commensal pathway and that repeated admixtures between the domesticated lineage and its wild ancestors, the grey wolves (*Canis lupus*), were probably an inherent character of the whole process [1–3]. However, hybridization may still occur in the wild through mating of wolves with escaped or feral dogs [4]. This may cause the introgression of "domestic" alleles into the natural populations [5], giving rise to novel conservation issues [6]. On the other hand, human-controlled hybridization of dogs with their wild ancestors has been repeatedly performed in captivity and gave rise to recently commercialized Wolfdog breeds such as the Saarloos Wolfdog, the Lupo Italiano, the Kunming Wolfdog and the Czechoslovakian Wolfdog [7].

The latter originated from first-generation German Shepherd (GS) *x* Carpathian wolf (CW) hybrids (F1) obtained in 1958 as a military project in the former Czechoslovakia [8]. However, backcrosses with dogs provided individuals that were less aggressive and easier to train than F1 and F2 hybrids. Therefore, through the subsequent crossings carried out between 1958 and 1983, the genomes of two males and two females of CW founders were deeply introgressed into the gene pool of GS, resulting in the Czechoslovakian Wolfdog (CSW) breed. The initial aim of the breeders was to select hard-working dogs for military purposes, to guard mountainous borders of the former Czechoslovakia during the Cold War, by improving their health, vitality, endurance and sensory abilities, as night vision. In this first phase, animals with intermediate wolf-dog phenotypes were not removed. While overspecialization is typical in many modern dog breeds, CSW breeders sought to balance this trend by adding characteristics of their wild progenitor. Animals that kept dog behavioral traits such as controllability together with a wolf phenotype were appreciated. In the second phase, especially after official approval of the breed standard by the Fédération Cynologique Internationale (FCI) in 1989, selection for wolf-like phenotypes dominated and CSW were kept as civilian dogs with similar utilization as GS. In 2012, the breed numbered 168 adult females and 170 adult males officially registered in the Czech Republic. However, although the number of CSW continues to increase all over the world and the breed is currently getting more and more diffuse with a relevant economic impact among breeders, only a few scientific studies describing the standard of the breed are available so far [4,9,10].

In this paper, we aimed to i) quantify the proportions of parental wolf and dog genomes retained in the CSW after 25 years of backcrossing by comparing the current breed’s genetic data with genotypes sampled from their ancestral founder populations, that is, the wild Carpathian wolves and the domestic German Shepherd dogs; ii) ascertain parameters of variation; (iii) assess the role of hybridization with wolves as a potential compensation for the loss of genetic variation; and iv) study the potential role of artificial selection (genetic hitchhiking) on the studied loci.

## Materials and Methods

### Sampling

In total, 79 individuals of CSW, 20 individuals of GS and 28 individuals of CW were analyzed. Cheek swab samples of CSW and GS were collected during dog shows in the Czech Republic. Only animals in healthy condition with permission and assistance of the owners were sampled, with every effort made to minimize their stress. One individual per litter was analyzed to avoid biases in genetic variability measurements. Wolf samples consisted of 25 non-invasive stool samples collected in the western Carpathians (*N* = 22), or obtained from the Prague Zoo (*N* = 3), and three tissue samples from Slovakia. Wolf stool samples were collected by Friends of the Earth organization (FoE CZ), which is monitoring the wolf population in the Carpathian Mountains. There are no restrictions for the use of stool samples in the Czech Republic. In Slovakia, FoE CZ has permission to collect non-invasive samples of wolves, issued by Regional Office Trenčín, Department of Environment, No. OU-TN-OSZP1-2014/49/3475.

The three tissue samples were derived from wolves that were legally culled during the open hunting season (November 1^st^–January 15^th^) in Slovakia within a quota set by the local authorities, in conformity with regulation No. 344/2009 Coll. The wolves were shot during individual patrols or collective hunts. The use of poisoned bait or leg-hold traps is strictly forbidden according to hunting law. All hunters had permission for hunting, and we confirmed that the culls were reported before quota fulfillment. No animals were sacrificed for the purposes of this study. Our laboratory has approval (No CZ 11712934) to storage and use of animal material according to § 48(1)(i) of Act No 166/1999 concerning veterinary care and amending certain related laws, as amended, pursuant to Article 17(1) of Regulation of the European Parliament and of the Council (EC) No 169/2009 and Article 27(1) of Commission Regulation (EU) No 142/2011. Tissue and stool samples were stored at −20°C in 10 volumes of 95% ethanol. Saliva samples were dry-stored.

### DNA extraction, sequencing and genotyping

Genomic DNA was extracted using the Genomic DNA Kit (Geneaid Biotech Ltd., New Taipei, Taiwan) for tissue and saliva or the QIAamp Stool Mini Kit (Qiagen Inc, Hilden, Germany) for stool samples. DNA from stool samples was extracted, amplified, and genotyped in separate rooms reserved for low-template DNA samples, under sterile ultraviolet laminar flow hoods, following a multiple-tube protocol [11] including both negative and positive controls.

Samples were thus genotyped based on the following: i) the Amelogenin gene (to sex individuals); ii) 39 autosomal microsatellites (to reconstruct individual genetic profiles) and four sex-linked microsatellites (Y-STR, to identify paternal haplotypes); and iii) the hypervariable part of the mtDNA control region (to determine maternal haplotypes).

Genotyping of the Amelogenin gene, 39 autosomal and four Y-linked microsatellites was performed as described in Randi et al. [4].

Amplifications were replicated twice for tissue and salivary samples and from four to eight times for fecal material. Allele sizes were manually scored in GeneMarker v.1.85 (www.softgenetics.com) and binned using raw size in Autobin (http://www4.bordeaux-aquitaine.inra.fr/biogeco/Ressources/Logiciels/Autobin).

Genotyping errors such as large alleles dropout, stuttering or null alleles were tested through Markov chain Monte Carlo (MCMC) simulations of expected allele-size differences using 1000 randomizations in Micro-Checker [12].

The hypervariable domain of the mtDNA control region was amplified using polymerase chain reaction (PCR) according to Vilà et al. [13]. Sequences were aligned using CLUSTAL W [14] implemented in BIOEDIT [15]. Identical haplotypes were collapsed in DNASP 5 [16] and were compared with the GenBank database using the megablast algorithm.

### Molecular data analysis

Genetic diversity measurements such as the mean number of different alleles per locus (*N*_*A*_), mean number of effective alleles per locus (*N*_*E*_), expected (*H*_*E*_) and observed (*H*_*O*_) heterozygosity, estimations of the inbreeding coefficient (*F*_*IS*_) and allelic richness with correction to equal sample size (*A*_*R*_) were computed in FSTAT 2.9.3.2 [17]. FSTAT uses rarefaction to standardize sample size of allelic richness to the *N* of the smallest group in the data set, which is 20 in this study. The number of private alleles (*N*_*P*_) was determined in GenAlEx 6.5 [18]. Deviations from Hardy-Weinberg equilibrium (HWE) and Linkage Equilibrium (LE) were tested in GenePop 4.0 [19], using exact tests and MCMC simulations with 100 batches of 1000 iterations. Factorial correspondence analysis (FCA) was performed in Genetix 4.05.2 [20]. The Bayesian clustering method [21] implemented in the program STRUCTURE 2.3 [22] was used with an admixture model and correlated allele frequencies to detect substructure in the data, assign individuals to clusters and identify potentially admixed genotypes. The optimal number of clusters (*K*) was set by running the program from *K* = 1 to *K* = 5, with 10 repetitions of 1 000 000 MCMC chain steps after a burn-in period of 100 000 steps for each *K*. STRUCTURE results were visualized in STRUCTURE HARVESTER [23] implementing the method of Evanno et al. [24]. Graphical output was performed in DISTRUCT 1.1 [25].

Contemporary effective population size (*Ne*) and 95% confidence intervals (CI) for CSW were estimated using, as single-sample estimator, a bias-corrected version of the linkage disequilibrium method [26] as implemented in the software NeEstimator v.2.0 [27]. This method uses multilocus diploid genotypes from a given population to obtain precise estimates of *Ne* with non-overlapping generations by using 10–20 microsatellite loci (5–10 alleles/locus) and samples of at least 25–50 individuals, if the effective population size is less than approximately 500 [28]. NeEstimator was run using the 79 CSW and considering a *P*_*Crit*_ value (for screening out rare alleles) of 0.02, which was recommended as the value ensuring the most precise and less biased results when working with microsatellites [27].

The effects of a population bottleneck on genetic variability parameters (*N*_*A*_, *N*_*E*_, *H*_*E*_, *H*_*O*_) in CSW were simulated using the program BottleSim v.2.6 [29], aiming to understand how the demographic aspects of the breeding process that led to Czechoslovak Wolfdog has modified the genetic variability from the two source populations. Because the genotypes of founder individuals were not available, in order to have a representation of the genetic variability of the two source gene pools, we started from the genotypes of the 20 GS and the 28 CW to simulate genotypes after 25 generations of mating using the various parameters, including diploid, multilocus, variable population size, non-overlapping generations, random mating system, eight years of expected longevity, age at reproduction of 1 year and sex ratio 1/1.

The simulated genotypes obtained after 25 generations were used to compare their genetic variability through time and with that of a real CSW population. Finally, *Ne* was recalculated in NeEstimator to detect if artificial selection might have caused a loss of heterozygosity or changes in allele frequencies in any significant way.

## Results

The alignment of 79 mtDNA sequences from CSW individuals showed the occurrence of only two distinct haplotypes. Twenty two dogs carried CSWA and 57 carried CSWB haplotypes, which differed by six mutations from each other [GenBank: KJ776748 and KJ776749]. Analysis in GenBank showed that both mtDNA haplotypes found in the CSW were shared with other domestic breeds but not with wolves.

Y-linked microsatellite variability analyses showed the presence of only two haplotypesin CSW, one shared with GS and one private (Table 1).

**Table 1:** Distribution of the Y-linked microsatellite haplotypes as named by Randi et al. (2014). For all haplotypes, the alleles of each locus are listed.

						Population	
Y-haplotypes	MSY34A	MSY34B	MSY41A	MSY41B	GS (11)	CSW (32)	CW (12)
YH01	168	177	113	118	0	21	0
**YH05**	170	175	113	126	**11**	**11**	**0**
YH11	172	175	113	126	0	0	5
YH16	174	173	113	122	0	0	1
YH31	174	173	113	126	0	0	5
YH49	172	175	113	124	0	0	1
Private haplotypes					0	1	4

GS—German Shepherds, CSW—Czechoslovakian Wolfdogs, CW—Carpathian wolves. In parentheses the number of individuals is reported for each group.

All the samples, including the 25 non-invasive samples, provided distinct multilocus genotypes at autosomal microsatellite loci. The biparental microsatellite dataset did not show any significant presence of genotyping errors after Bonferroni corrections. All of the 39 autosomal microsatellites were polymorphic in CSW with a total of 188 alleles. Mean *N*_*A*_ across all loci was 4.82 and ranged from 2 to 8 alleles per locus. The total number of *N*_*P*_ was 20, and none of the private alleles were shared with CW or GS. Average *F*_*IS*_ was 0.004, mean *H*_*O*_ = 0.5420, mean *H*_*E*_ = 0.5409 and mean *A*_*R*_ = 3.751 in CSW (Table 2). While CW carry the highest number of *N*_*P*_ (*N*_*P*_ = 60) and their *A*_*R*_ is the highest among the studied groups (*A*_*R*_ = 4.626), they have the largest differences between *H*_*E*_ (*H*_*E*_ = 0.6404) and *H*_*O*_ (*H*_*O*_ = 0.691); thus, their *F*_*IS*_ (*F*_*IS*_ = 0.069) is slightly elevated compared to GS and CSW (Table 2).

**Table 2:** Genetic variability in the three analyzed groups at 39 autosomal microsatellite loci.

Group	*N*	*N*_*A*_	*N*_*E*_	*P*_*N*_	*A*_*R*_	*H*_*O*_	*H*_*E*_	*F*_*IS*_
CSW	79	4.82	2.39	20	3.751	0.5420	0.5409	0.004
GS	20	3.90	2.26	11	3.709	0.5026	0.4921	0.005
CW	28	5.08	3.13	63	4.626	0.6091	0.6404	0.069

CSW—Czechoslovakian Wolfdogs, GS—German Shepherds, CW—Carpathian wolves.

Number of analyzed individuals (*N*), mean number of alleles across all studied loci (*N*_*A*_), mean number of effective alleles per locus (***N***_***E***_), total number of private alleles (*P*_*N*_), mean allelic richness corrected by sample size (*A*_*R*_), observed (*H*_*O*_) and expected (*H*_*E*_) heterozygosity, inbreeding coefficient (*F*_*IS*_).

**Fig 1:**
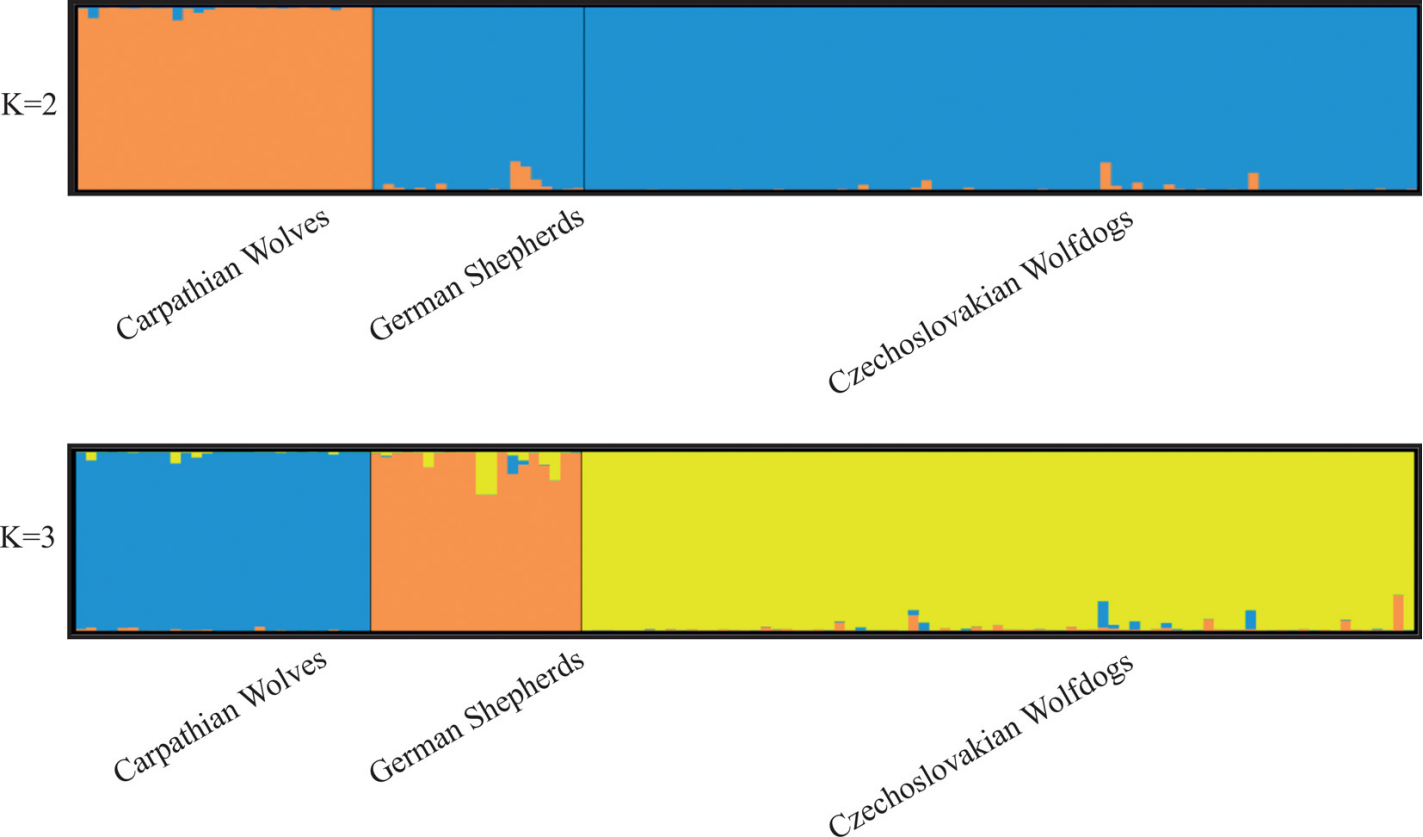
Bayesian clustering analysis of the three populations obtained by STRUCTURE. Each individual is represented by one vertical bar that is divided into segments representing the proportion of memberships to the respective populations. The results are displayed for two (*K* = 2) and three (*K* = 3) suggested clusters.

**Fig 2:**
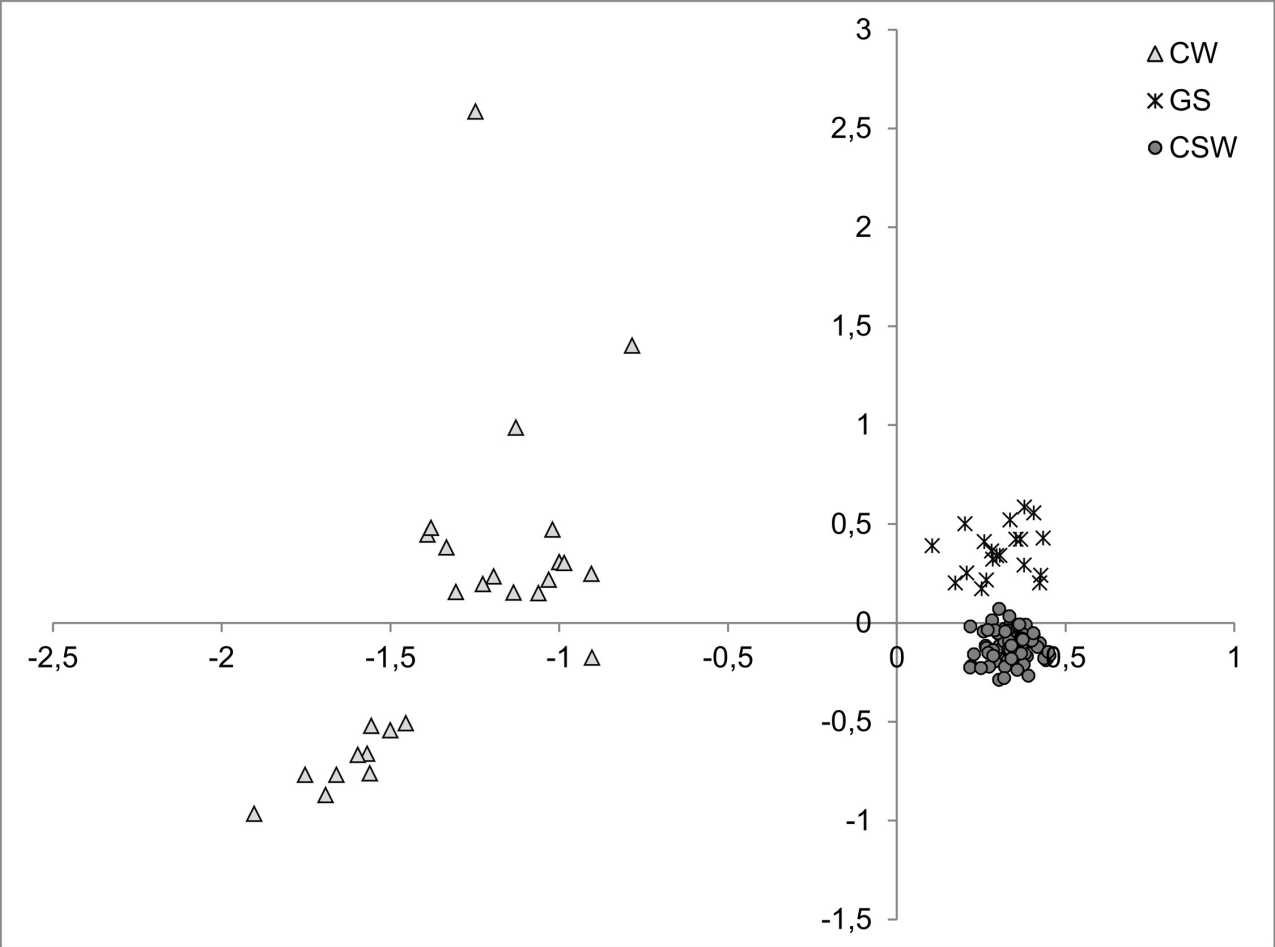
A two-dimensional plot of the factorial correspondence analysis performed in Genetix. CW = Carpathian wolves, GS = German Shepherds, CSW = Czechoslovakian Wolfdogs. NeEstimator showed a high concordance between the results obtained considering *P*_*Crit*_ = 0 and *P*_*Crit*_ = 0.02, from which the effective CSW population size was *Ne* = 76.5 (95% CI: 68.2–86.5) and *Ne* = 82.9 (95% CI: 72.3–96.4), respectively.

Czechoslovakian Wolfdogs did not show significant deviations from HWE and LE. The best-supported number of clusters in STRUCTURE was *K* = 2, separating pure wolves from both dog breeds (mean estimated membership of population to the assigned cluster (*Q*_i_) was 0.993). However, at *K* = 3, all individuals were correctly assigned to their own breed (CSW or GS) or wolf clusters with *q*_*ì*_ values > 0.80 (mean *Q*_*i*_ was 0.981) and any internal substructure was detected among CSW (Fig 1). Details of Bayesian analysis in STRUCTURE are shown in the appendix (S1 Table). Factorial correspondence analysis clearly separated the three studied groups. The position of the CSW individuals in the plot is not intermediate between the parental groups of GS and CW, but it is closer to GS (Fig 2).

The scenario obtained using BottleSim showed a slight reduction of variability indexes through generations, as expected because of inbreeding and drift due to bottleneck demography of the breed. Specifically we observed a reduction in the heterozygosity: *H*_*O*_ from 0.6944 to 0.5865 and *H*_*E*_ from 0.6095 to 0.5812. Such a reduction also appears when genetic variability is compared to the real CSW population analyzed (see S2 Table) as CSW showed a higher *N*_*A*_ (*N*_*A*_ = 4.74 vs. 3.61) but a lower *N*_*E*_ (*N*_*E*_ = 2.38 vs. 2.69) and heterozygosity values (*H*_*O*_ = 0.54 vs. 0.59, *H*_*E*_ = 0.54 vs. 0.58) compared with simulated data (25th generation).

When we used the 339 simulated genotypes obtained after 25 generations of mating in NeEstimator, the effective CSW population sizes we observed, considering *P*_*Crit*_ = 0 and *P*_*Crit*_ = 0.02, were *Ne* = 94.4 (95% CI: 87.8–101.6) and *Ne* = 92.8 (95% CI: 86.0–100.2), respectively, which are values, that are slightly higher than those obtained with real data. This difference could suggest a change in the maintenance of the genetic variation because of the non-random mating applied in the origin of the breed.

## Discussion

### Genetic variation and proportions of ancestral genomes

In this study, we obtained estimates of genetic diversity parameters, including mean number of alleles per locus (*N*_*A*_), mean number of effective alleles per locus (***N***_***E***_), number of private alleles (*N*_*P*_), allelic richness (*A*_*R*_), expected (*H*_*E*_) and observed (*H*_*O*_) heterozygosity, inbreeding coefficient (*F*_*IS*_), and Hardy-Weinberg equilibrium (HWE) deviations, in the recent Czechoslovakian Wolfdog breed that were compared to those obtained from the breed’s ancestral founder populations, the wild Carpathian wolves and the domestic German Shepherd dogs.

Our results show a high proportion of dog genome in CSW, which is in agreement with the origin of the breed and data from previous studies [10]. For example, the position of CSW in the FCA is closer to GS, as expected as a consequence of the 25 years of backcrossing. The lack of wolf mtDNA haplotypes indicates the loss of these variants during the lineage sorting acting in the Wolfdog pedigree. The same evidence also seems to be associated with the Y-chromosome, as we found that none of the two Y-haplotypes detected in the 32 CSW males we analyzed for this study were shared with CW. Using the same panel of four Y-linked microsatellites, Čílová et al. [30] found that some of the CSW males still carry one Y-haplotype of wolf origin, although they did not report the frequency of this haplotype in the CSW population. The different CSW and reference CW samples used in the analyses could be the reason why we did not detect Y-haplotypes derived from wolves. Moreover, the moderate number of microsatellite markers used in our analyses may not reflect the whole genome variability of the breed.

### The role of demography and hybridization in shaping the genetic architecture of the breed

Analyses in BottleSim indicated that neutral processes acting in small populations, such as the founder effect and genetic drift [31], have changed the genetic composition of CSW. This is in agreement with the relatively low number of founding individuals of this breed. The loss of genetic variation may cause the overrepresentation of some deleterious alleles in many dog breeds including CSW and GS [32], causing genetic diseases such as dwarfism [33] and degenerative myelopathy [34]. On the other hand, populations of moderate size may still possess some degree of evolutionary potential [35]. Among proximate mechanisms responsible for the positive effects of bottleneck, purging of deleterious alleles was also described [36]. However, the F_IS_ value was relatively low in CSW compared with other purebred dog breeds [37]. Recent simulation and empirical studies showed that diverse life history traits, including mating patterns and overlap of generations, may influence the effect of bottleneck on diversity patterns [38]. The loss of genetic variation may also be caused by rapid post-bottleneck recovery [39]. However, as the life history traits in CSW are similar to other breeds and the population size remained relatively low during the breed’s history, the compensation for the expected founder effect and genetic drift could be ascribed to the effects of outbreeding that are related to the introgression of wolf alleles. The high variability expected in hybrids, deduced from assumptions of Mendelian inheritance, could cause some hybrids to be far from parental phenotypic optima and start evolutionary trajectories that are divergent from the parental forms [40]. This may also explain the position of CSW in FCA, which is not at the center between both parental populations.

### Artificial selection and genetic hitchhiking

The position of the breed in cluster analysis may also be influenced by other factors. After obtaining admixed genotypes between shepherd dogs and their wild ancestors, the population of captive Wolfdogs experienced artificial selection that aimed to keep wolf-like phenotypes while preserving dog behavior and the inherent preference of other characteristics connected with domestication. These traits are associated with many genomic regions, related to, for example, morphology, physiology and behavior [1]. If some of the examined neutral loci were linked to these genes under selection, their allele frequency could be changed by selective sweep or background selection [41]. For example, coat color, which is in strong artificial selection in dogs including CSW, is controlled by more than 300 known genetic loci and 150 known genes in mammals [42]. Considering physiological traits, comparison of wolf and dog genomes provided evidence that dogs, for example, adapted to a starch-rich diet by a higher production of amylase and maltase-glucoamylase [1]. Last but not least, evolutionary novelties accompanying domestication also include complex changes in social behavior [43], which in dogs enabled social interactions with humans including cooperation, social learning and communication [1,44,45]. Controllability was one of the key factors targeted during the breeding process of CSW.

## Conclusions

In the present study, we have described the impact of processes such as hybridization between dogs and wolves, population bottlenecks and artificial selection on the genetic diversity patterns in the Czechoslovakian Wolfdog. It seems that hybridization with the dog’s wild ancestors may have compensated for the loss of genetic variation caused by bottleneck demography. The phase of artificial selection applied to mosaic wolf *x* dog hybrid genomes was unique for many reasons, especially the following: i) it was very fast compared to the natural domestication process, which possibly lasted thousands of years, and therefore, selection pressures were presumably intense; and ii) it included direct selection for morphological traits, whereas during the first domestication phase, the main selective pressures presumably acted on behavioral (or physiological) traits, with phenotypic variants originating as by-products of these changes [43]. To conclude, the mode and direction of selection acting on particular traits were different compared to the domestication process (e.g., preferring phenotypes of wild ancestors), which enables us to study particular modules of domestication in different contexts. Due to these unique evolutionary pathways, wolfdogs represent interesting models for studying the outcomes of interactions between socialized and wild canid genomes and the role of processes accompanying animal adaptations to the human environment.

## Supporting Information

S1 Table Parameters of analysis in STRUCTURE for K1-K5. (XLSX)

S2 Table Genotypes and population size (N) after 25 generations of mating simulated using program BOTTLESIM. (XLS)
